# Identification and validation of signature for prognosis and immune microenvironment in gastric cancer based on m6A demethylase ALKBH5

**DOI:** 10.3389/fonc.2022.1079402

**Published:** 2023-01-06

**Authors:** Tiannan Ji, Xiaohui Gao, Dan Li, Siyuan Huai, Yajing Chi, Xian An, Wenyu Ji, Siming Yang, Jianxiong Li

**Affiliations:** ^1^ Medical School of Chinese PLA, Beijing, China; ^2^ Department of Radiotherapy, Senior Department of Oncology, the Fifth Medical Center of PLA General Hospital, Beijing, China; ^3^ Department of Clinical Medicine, Graduate School of Hebei North University, Zhangjiakou, Hebei, China; ^4^ School of Medicine, Nankai University, Tianjin, China

**Keywords:** ALKBH5, gastric cancer, immune cell infiltration, m6A, prognosis

## Abstract

**Background:**

N6-methyladenosine (m6A) RNA regulators play important roles in cancers, but their functions and mechanism have not been demonstrated clearly in gastric cancer (GC).

**Methods:**

In this study, the GC samples with clinical information and RNA transcriptome were downloaded from The Cancer Genome Atlas database. The different expression genes were compared by the absolute value and median ± standard deviation. Samples with complete information were randomly divided into a training dataset and a test dataset. The differential expression genes (DEGs) between *ALKBH5*-low and *ALKBH5*-high subgroups were identified in the training dataset and constructed a risk model by Cox and least absolute shrinkage and selection operator regression. The model was testified in test datasets, overall survival (OS) was compared with the Kaplan–Meier method, and immune cell infiltration was calculated by the CIBERSORT algorithm in the low-risk and high-risk subgroups based on the model. The protein levels of ALKBH5 were detected with immunohistochemistry. The relative expression of messenger-ribonucleic acid (mRNA) was detected with quantitative polymerase chain reaction.

**Results:**

*ALKBH5* was the only regulator whose expression was lower in tumor samples than that in normal samples. The low expression of *ALKBH5* led to the poor OS of GC patients and seemed to be an independent protective factor. The model based on *ALKBH5*-regulated genes was validated in both datasets (training/test) and displayed a potential capacity to predict a clinical prognosis. Gene Ontology analysis implied that the DEGs were involved in the immune response; CIBERSORT results indicated that ALKBH5 and its related genes could alter the immune microenvironment of GC. The protein levels of ALKBH5 were verified as lowly expressed in GC tissues. *SLC7A2* and *CGB3* were downregulated with *ALKBH5* knockdown.

**Conclusions:**

In this study, we found that ALKBH5 might be a suppressor of GC; ALKBH5 and its related genes were latent biomarkers and immunotherapy targets.

## 1 Introduction

Gastric cancer (GC) is one of the most threatening diseases worldwide, which gave the fifth incidence and fourth mortality in cancers in 2020 (1). An appropriate option of surgical resection is the only strategy to treat early disease. However, due to inconspicuous early symptoms, patients are always diagnosed in advanced stages; thus, follow-up chemotherapy/targeted therapy and immunotherapy are needed. Unfortunately, the efficiency is limited because of late detection and the lack of therapeutic targets (2); therefore, it is urgent to find novel approaches to improve the cure rate.

N6-methyladenosine (m6A) is the most abundant modification on mRNAs of eukaryotes (3); it is a highly conserved and dynamic reversible process regulated by the m6A methyltransferases (writers) or demethylases (erasers) that add or remove the m6A sites in mRNAs and recognized by m6A-binding proteins (readers) (4, 5). Writers mainly include METTL3, METTL14, WTAP, VIRMA/KIAA1429, RBM15/15B, and ZC3H13 (6–9). Erasers mainly include fat mass and obesity–associated protein (FTO) and ALKBH5 (4, 10). Readers mainly include YTHDF1/2/3, YTHDC1/2, IGF2BP1/2/3, HNRNPC, and HNRNPA2B1 (11–16). m6A regulators participate in various physiological and pathological processes in tumor occurrence and development, acting as promoters or inhibitors. For instance, METTL3 accelerated the maturation of pri-miR221/222, resulting in the reduction of PTEN, which ultimately leads to the proliferation of bladder cancer (17). In breast cancer, FTO degraded BNIP3 through the demethylation of m6A in the 3'Untranslated Regions (3'UTR), leading to tumorigenesis and a poor prognosis (18). Furthermore, METTL14 attenuated the proliferation and migration ability of renal cell carcinoma cells by decreasing the expression of long non-coding RNA (lncRNA) nuclear-enriched abundant transcript 1 (NEAT1_1) (19). In glioblastoma, YTHDF2 tended to be a therapeutic target; it could stabilize the transcripts of MYC and therefore regulate glucose metabolism in glioblastoma stem cells (GSCs) (20).

In GC, there are a lot of important functions induced by m6A regulators and they participate in various oncogenic signaling pathways as well. Wang et al. demonstrated that the level of METTL3 was significantly elevated in GC tissues and associated with a poor prognosis. It promoted the tumorigenesis and metastasis of GC by stimulating the m6A modification of Hepatoma Derived Growth Factor (HDGF) mRNA and then activated GLUT4 and ENO2 expression (21). Yue et al. also revealed that METTL3 facilitated GC by regulating the m6A level of *ZMYM1*, which could increase the expression of E-cadherin and promote the epithelial–mesenchymal transition process (22). Huo et al. found that METTL3 accelerated the development of GC by the METTL3-SPHK2-KLF2 axis (23). Furthermore, Pi et al. illustrated that YTHDF1 promoted the translation of a key Wnt receptor frizzled7 (FZD7), leading to the hyperactivation of the Wnt/β-catenin pathway and the promotion of gastric carcinogenesis (24). Chen et al. found that YTHDF1 also facilitated the tumorigenesis and metastasis of GC by promoting USP14 protein translation in an m6A-dependent manner (25). FTO was verified to lead to the metastasis of GC by decreasing the m6A level and expression of ITGB1 (26); meanwhile, Yang et al. demonstrated that it promoted the development of GC by the FTO-m6A-MYC axis (27). These regulators also executed functions by shaping lncRNA. Lv et al. have revealed that the m6A levels of lncRNAs were changed in GC by bioinformatic manners and the difference might have a predictive value (28). Moreover, Hu et al. found that LINC01320 could be elevated by METTL14 and promotes the proliferation, migration, and invasion of GC *via* the miR495-5p/RAB19 axis (29).

ALKBH5 is short for alkylation repair homolog protein 5, a demethylase of m6A. The differential expression and regulatory functions of ALKBH5 in multiple cancers have been reported. In acute myeloid leukemia (AML), ALKBH5 was positively regulated by KDM4C and the high level of ALKBH5 increased the stability of AXL (AXL receptor tyrosine kinase), thus promoting leukemia stem cells (30). In glioblastoma, ALKBH5 promoted tumorigenesis and development by demethylating *FOXM1* nascent transcripts, which led to an enhanced expression of FOXM1 (31). In pancreatic cancer, Guo et al. elucidated that ALKBH5 reduced tumor proliferation, migration, and invasion by activating PER1 (period circadian regulator 1) in an m6A-dependent manner (32), Tang et al. declared that ALKBH5 also increased the expression of WIF-1 (Wnt inhibitory factor 1) and inhibited the Wnt pathway to suppress these tumor features (33). In non-small cell lung cancer (NSCLC), ALKBH5 inhibited tumor growth and metastasis by decreasing YAP activity and regulating the miR-107/LATS2 axis in an HuR (a RNA binding protein)-dependent manner (34). However, the role of ALKBH5 in GC was conflicted and obscure. Ge et al. demonstrated that there was a lower expression of ALKBH5 in GC peripheral blood compared with healthy controls and it might be a protective gene for GC patients (35). Meanwhile, Zhang et al. elucidated that ALKBH5 promoted GC invasion and metastasis by the demethylation of *NEAT1* lncRNA (36). According to the present situation, the expression, function, and mechanism of ALKBH5 in GC are still worthy to be investigated and validated.

**Figure 1:**
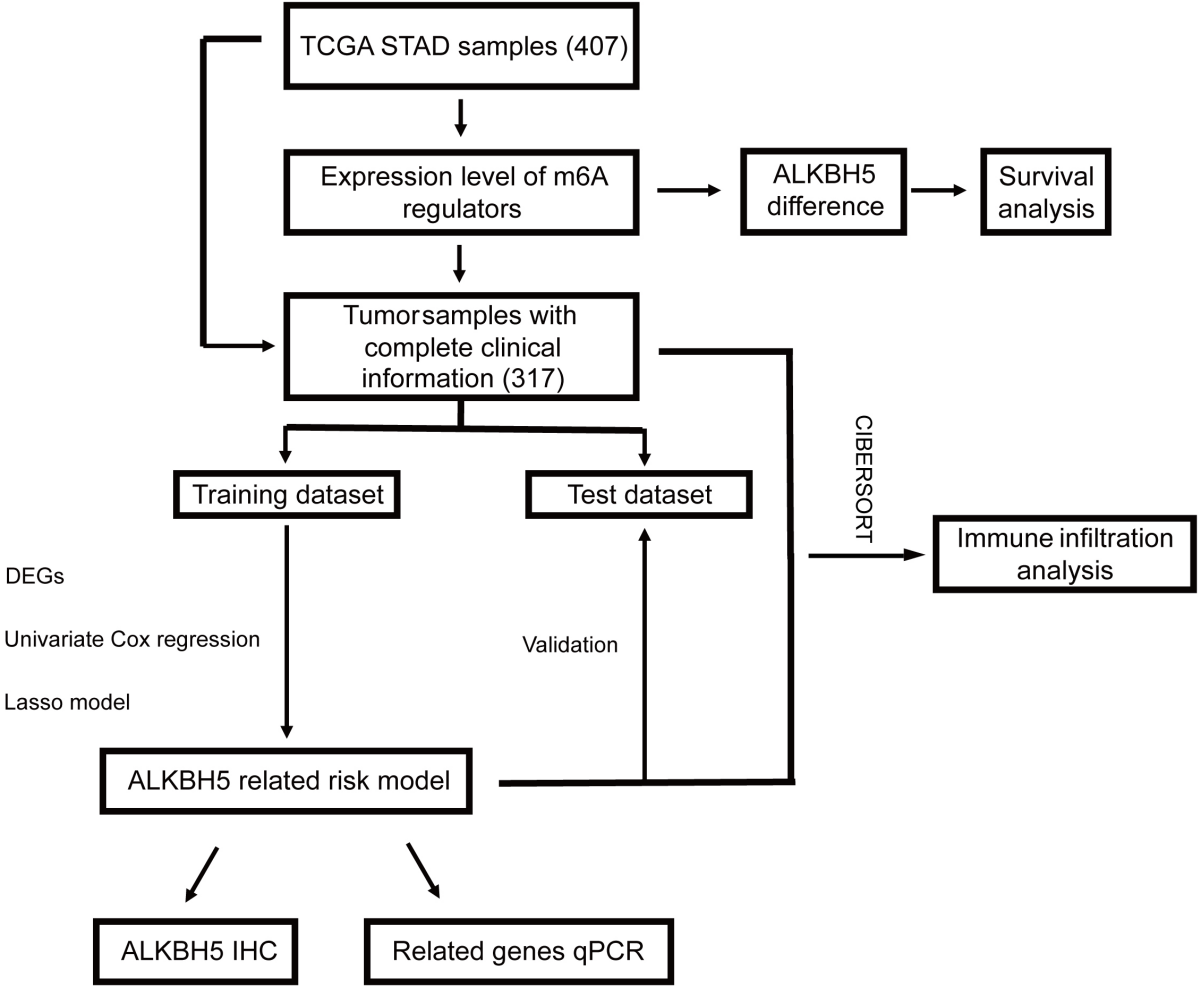
Workflow chart of our study. STAD, stomach adenocarcinoma.

In this study, as shown in the workflow chart (**Figure 1**), we downloaded and comprehensively analyzed stomach adenocarcinoma (STAD) samples from The Cancer Genome Atlas database (TCGA, https://portal.gdc.cancer.gov/). Comparing the m6A regulator genes’ expression levels in the tumor group and normal group and investigating their relationship, we found that the expression of *ALKBH5* was lower in tumor samples than normal ones and a high level of *ALKBH5* led to a better prognosis. Then, the whole cohort was randomly divided into a training dataset and a test dataset. A risk model and a nomogram model for predicting the prognosis of GC patients were constructed in the training dataset and validated in the test dataset. Furthermore, Gene Ontology (GO) analysis suggested that the differential expression genes (DEGs) might be enriched in immune defense; thus, we computed the immune cell infiltration in all of the samples by the CIBERSORT algorithm and the proportion of these cells in ALKBH5-low/high and risk-low/high subgroups indicated that *ALKBH5*-high and risk-low tissues were infiltrated with more effective immune cells, respectively. Finally, we checked the expression levels of ALKBH5 between GC tumor tissues and adjacent normal tissues with immunohistochemistry (IHC) and the relative expression levels of genes used to construct the risk model when *ALKBH5* was knocked down with real-time quantitative PCR (RT-qPCR). Coinciding with our model, the protein level of ALKBH5 in tumor tissues was lower than that in normal tissues, and some tumor suppressor genes were down expressed when ALKBH5 was knocked down. The findings in this study indicate that ALKBH5 may play a suppressor role in GC and its related genes act as latent predictive biomarkers.

## 2 Material and methods

### 2.1 Data acquisition and arrangement

The major clinical information and RNA transcriptome dataset (FPKM-UQ and counts) of GC samples and normal samples were downloaded from TCGA (https://portal.gdc.cancer.gov/). STAD is a major type of GC, and the number of other types of gastric cancer is too small to analyze; thus, we chose STAD samples for our study. A total of 407 samples were downloaded, including 375 primary tumor samples and 32 normal samples. When analyzing the prognosis of target genes or different clusters, samples without complete clinical information were wiped out and the remaining ones were randomly divided into a training dataset and a test dataset. The transcriptome data for validating the expression levels of ALKBH5 were downloaded from Gene Expression Omnibus (GEO, https://www.ncbi.nlm.nih.gov/geo/).

### 2.2 Bioinformatic analysis

#### 2.2.1 Comparison of the expression levels of m6A regulator genes

The different expression levels of m6A regulator genes were compared by the absolute value and median ± standard deviation (SD), and the results were displayed by R software using “pheatmap” and “ggplot2” packages. We set overall survival (OS) as the major criterion for the evaluation of different cohorts’ death risk. It means from randomization to the time of death from any cause. For subjects who were lost to follow-up before death, the time of the last follow-up is usually calculated as the time of death. The OS between *ALKBH5*-low/*ALKBH5*-high and low-risk/high-risk subgroups, as well as univariate Cox regression and multivariate Cox regression, was conducted by the “survival” package.

#### 2.2.2 Construction of the risk model

The “edgeR” package was chosen to identify the differential expression genes (DEGs) between *ALKBH5*-low and *ALKBH5*-high subgroups; the relationship of these genes and the samples’ OS was evaluated with univariate Cox regression. After screening out genes with significant differences, the “glmnet” package was used to conduct least absolute shrinkage and selection operator (LASSO)–penalized Cox regression. According to the result of LASSO regression, the risk model was constructed by the following prognosis formula: risk score = where expi represented log2 (gene expression + 1) and coefi represented the coefficient of each gene. The model was applied in each sample in the training/test dataset, and the median value was set as the cut-off; thus, samples in both datasets were divided into the low-risk subgroup and high-risk subgroup, respectively. Furthermore, a sequence-based RNA adenosine methylation site predictor (SRAMP) database was used to search for the prediction scores of possible adenosine methylation sites on ALKBH5-related genes in the risk model.

#### 2.2.3 Validation of the risk model

To validate the model and roughly get its predictive efficacy in clinical GC patients, in the training dataset and test dataset, we compared the OS curves with the Kaplan–Meier method and explored the distribution of samples’ vital status and survival time according to the risk score with dot plots. Furthermore, univariate/multivariate Cox regression and receiver operating characteristic (ROC) curves were conducted. The ROC curves were accomplished by the “pROC” package. Thereafter, to further clarify the prognosis value of the model, the OS in the various clinical features cohort (including gender, age, stage, grade, and T/N stages) between the low-risk and high-risk subgroups were compared in the two datasets.

### 2.3 Nomogram

A nomogram for predicting the prognosis of GC was built with the “rms” package in the training dataset, and the calibration curves were conducted subsequently. Then, the nomogram was checked in the test dataset and decision curve analysis (DCA) was performed by the “rmda” package.

### 2.4 Analysis of immune cell infiltration

To seek the potential function of the DEGs, Gene Ontology (GO) analysis was operated with the “clusterProfiler” package (37, 38). Meanwhile, the CIBERSORT algorithm was used to calculate the 22 immune cell infiltration proportions of each sample. The different infiltration ratios among *ALKBH5*-low/*ALKBH5*-high and low-risk/high-risk subgroups were compared in the whole dataset.

### 2.5 Human tissue microarray and immunohistochemistry

A tissue microarray containing the slides of 90 GC tumor tissues and adjacent normal tissue was purchased from Outdo Biotech with ethical approval (Shanghai, China; HStmA180Su11). The protein level of ALKBH5 was determined by a semiquantitative IHC assay, using the anti-ALKBH5 antibody (Abcam, ab244296). The results of IHC were independently given stained scores by two independent observers. The criteria are as follows: 1) ≤25% of positively stained cells; 2) 25%–50% of positively stained cells; 3) 50%–75% of positively stained cells; and 4) ≥75% of positively stained cells.

### 2.6 Cell culture

Human GC cell lines (MGC-803 and HGC-27) were ordered from American Type Culture Collection (ATCC, Manassas, VA, USA). Each cell line was authenticated by measuring the short-tandem repeat (STR) DNA profiles. No contamination of mycoplasma was found in these cell lines. Both the two cell lines were cultured in DMEM (Gibco, NY, USA) containing 10% fetal bovine serum (FBS, Gibco, Grand Island, NY, USA) in a humidified atmosphere with 5% CO_2_ at 37°C.

### 2.7 siRNA and cell transfection

The siRNA targeting *ALKBH5* was designed and synthesized by RiboBio Co., Ltd. (Guangzhou, China). MGC-803 and HGC-27 cells were transfected with si-ALKBH5 using the riboFECT CP Transfection Kit (C10511-05) (Riobio, Guangzhou, China) according to the manufacturer’s protocol. The si-ALKBH5 sequence was -GCTGCAAGTTCCAGTTCAA-.

### 2.8 RT-qPCR

Total RNA was isolated with the TRIzol reagents (Life Technologies, Shanghai, China) according to the manufacturer’s instructions. There were 3 μg of total RNA reverse-transcribed into complementary deoxyribonucleic acid (cDNA) using the Vazyme HiScript III RT SuperMix for qPCR (+gDNA wiper) kit (R323-01, Nanjing, China). The transcript level of the specific gene was amplified with the Vazyme ChamQ Universal SYBR qPCR Master Mix (Q711-02) and was normalized to Glyceraldehyde-3-Phosphate Dehydrogenase (GAPDH). The primers were synthesized by BGI TECH SOLUTIONS (BEIJING LIUHE) CO., LIMITED (Beijing, China), and the sequences are listed as follows: GAPDH-Forward, -GGAGCGAGATCCCTCCAAAAT-, GAPDH-Reverse, -GGCTGTTGTCATACTTCTCATGG-; CA10-Forwar, -CTGTCCAGCCACTCAACAAC-, CA10-Reverse, -AGGTGGGATTCTTCTTGGCT-; SLC7A2-Forward, -GACCTTTGCCCGATGTCTGAT-, SLC7A2-Reverse, -AGCAGCGGCATAATTTGGTGT-; CGB3-Forward, -CCCGAGGTATAAAGCCAGGT-, CGB3-Reverse, -GTAGTTGCACACCACCTGAG-; C1QL2-Forward, -TCGGCAATCACTATGACCCC-, C1QL2-Reverse, -CGCATGAGGATGTGGTAGGT-; CGB8-Forward, -GCCTTCCTACACCCTACTCC-, CGB8-Reverse, -CCAGGAGGTTGTAGGATGCT.

### 2.9 Statistical analysis

R software version R-4.1.2 for windows (The R Foundation for Statistical Computing, Vienna, Austria), SPSS version 22.0 (SPSS Inc., Chicago, IL, USA), and GraphPad Prism version 8.0 for Windows (GraphPad Software, San Diego, CA, USA, www.graphpad.com) were used for data analysis. A t test was used to compare continuous variables; a chi-square test was used to compare categorical variables. Univariate and multivariate Cox regression were performed to identify independent prognostic factors for OS. The Kaplan–Meier method was used to conduct the OS in different groups, and the log-rank test was used for comparing the survival curves. A P-value <0.05 was considered statistically significant.

## 3 Results

### 3.1 The expression and relationship of m6A-related genes

**Figure 2:**
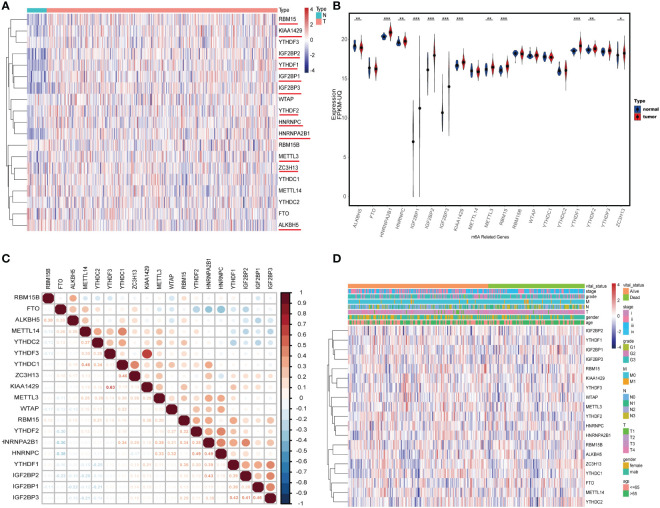
The landscape of N6-methyladenosine (m6A) regulator genes in gastric cancer (GC). **(A)** Heatmap of 19 m6A RNA regulator genes’ expression in GC. N: solid normal samples, T: tumoral samples. **(B)** The correlation coefficient of 19 m6A RNA regulators. **(C)** Violin plot visualizing the expression and distribution levels of 19 m6A regulators in GC. **(D)** Heatmap of 19 m6A RNA regulators’ expression in tumoral samples in different cohorts including age, gender, stage, grade, T, N, and M. *P < 0.05, **P < 0.01, and ***P < 0.001.

We first compared the expression levels of 19 m6A regulator genes between 375 primary tumor samples and 32 solid tissue samples; most of these genes showed higher expression in tumor samples than normal samples, including *HNRNPA2B1*, *HNRNPC*, *IGF2BP1*, *IGF2BP2*, *IGF2BP2*, *KIAA1429*, *METTL3*, *RBM15*, *YTHDF1*, *YTHDF2*, and *ZC3H13*, but the expression level of *ALKBH5* was opposite (**Figures 2A, B**). Then, the difference of *ALKBH5*’s expression was checked and verified in GEO dataset GSE29998; there was the same expression difference of *ALKBH5* in GSE29998 (**Figure S1**). The correlation coefficients of these regulators were detected with the Pearson method. The results demonstrated that, among the 19 regulator genes, *KIAA1429* and *YTHDF3* had the strongest correlation (R = 0.63); the top three genes related to *ALKBH5* were *RBM15B* (R = 0.30), *FTO* (R = 0.30), and *METTL14* (R = 0.25) (**Figure 2C**). Furthermore, the normal samples were wiped out, and the expression landscape in the tumor cohort was conducted to further see the relationship between them. In different clinical and pathologic cohorts including gender, age, stage, grade, T, N, and M, the distribution of these genes’ expression levels had differences (**Figure 2D**).

### 3.2 *ALKBH5* is a protective gene in GC and a risk model based on ALKBH5-related genes was conducted

**Figure 3:**
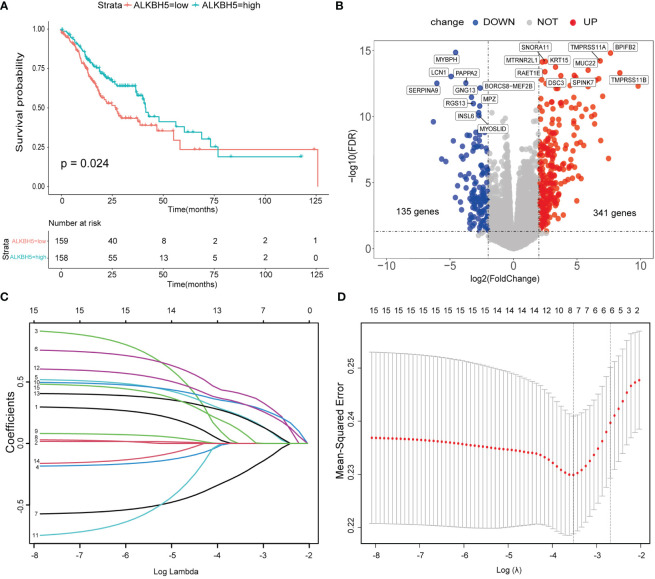
A risk model based on ALKBH5-related genes was conducted. **(A)** Overall survival (OS) of ALKBH5-low and ALKBH5-high subgroups. **(B)** The differential expression genes (DEGs) between ALKBH5-low and ALKBH5-high subgroups in the training dataset. Red points: upregulated genes in the ALKBH5-high subgroup, blue points: downregulated genes in *ALKBH5*-high subgroup. The top 10 upregulated genes and downregulated genes are also shown. **(C)** The least absolute shrinkage and selection operator (LASSO) coefficient of 15 significant DEGs (P< 0.05) in the univariate Cox regression model with all of the DEGs. **(D)** Selecting the best lambda parameters for the LASSO model.

To investigate the relationship between the regulators and GC patients’ OS, 58 tumor cases without complete clinical information were omitted; then, univariate and multivariate Cox regression were performed in the remaining 317 samples. Both univariate and multivariate Cox models indicated that *ALKBH5* might be an independent protective factor for GC patients. RBM15 and *YTHDF2* also showed the same hazard ratio as *ALKBH5*, but *ALKBH5* showed significance for both univariate and multivariate Cox regression (**Figures S2A, B**). Furthermore, we drew survival curves with the vital status data in *ALKBH5*-low and *ALKBH5*-high subgroups and compared them; the cut-off of groups was median values. The result indicated that the *ALKBH5*-low subgroup had a shorter OS than the *ALKBH5*-high subgroup with statistical significance (**Figure 3A**).

To better decode the roles of *ALKBH5* in GC, the remaining 317 samples were randomized into the training dataset (n = 159) and test dataset (n = 158); the baseline of the two datasets is displayed in **Table 1**. In the training dataset, the “edgeR” package was used to obtain DEGs between *ALKBH5*-low and *ALKBH5*-high subgroups with the following conditions: log Foldchange > 2, adjust-P-value <0.05 (**Figure 3B**, **Table S1**). Then, univariate Cox regression was conducted to investigate the relationship between the DEGs and the samples’ OS (**Table S2**). For those significant ones (P-value < 0.05), we performed the LASSO-penalized Cox regression (**Figures 3C, D**). Constructing the risk model with the prognosis formula in the method section, the risk score = 0.006188505 * *CA10* + 0.050823131 * SLC7A2 − 0.008562421 * *LINC02303* + 0.050382245 * *CGB3* + 0.042501742 * *C1QL2* + 0.00927148 *CGB8. All samples’ risk scores in both datasets were calculated with the formula; then, samples in each dataset were divided into low-risk and high-risk subgroups by the median value.

**Table 1:** The baseline of patients in the training dataset and test dataset.

Variables	Training Dataset (n = 159)	Test Dataset (n = 158)	P-value
Age (n%)			0.1956
≤65	64 (40.25%)	76 (48.10%)	
>65	95 (59.75%)	82 (51.90%)	
Gender (n%)			0.6118
Male	102 (64.15%)	96 (60.76%)	
Female	57 (35.85%)	62 (39.24%)	
Grade (n%)			0.7827
G1 and G2	57 (35.85%)	60 (38.00%)	
G3	102 (64.15%)	98 (62.00%)	
T (n%)			0.1457
T1–2	34 (21.38%)	46 (29.11%)	
T3–4	125 (78.62%)	112 (70.89%)	
N (n%)			0.8737
N0	49 (30.82%)	51 (32.28%)	
N+	110 (69.18%)	107 (67.72%)	
M (n%)			0.4975
M0	150(94.34%)	145 (91.77%)	
M1	9 (5.66%)	13 (8.23%)	

In addition, prediction scores in SRAMP (39) suggested that there were considerable adenosine methylation sites with very high confidence or high confidence, especially in *CA10*, *LINC02303*, and *C1QL2* (**Figures S3A-F**).

### 3.3 The risk model based on ALKBH5-related genes has strong association with clinical prognosis in GC

**Figure 4:**
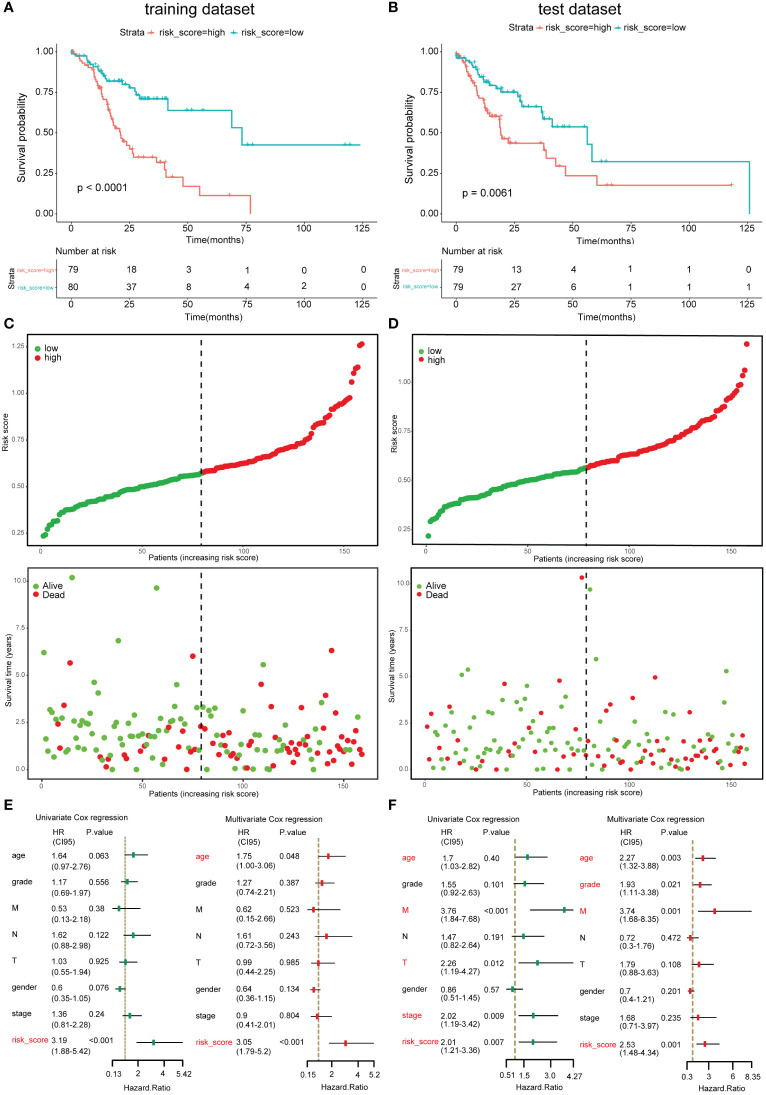
Validation of the risk model in the training dataset and test dataset. **(A, B)** Overall survival (OS) of the low-risk and high-risk subgroup in training dataset and test dataset. **(C, D)** The distribution of samples’ survival time and vital status according to risk scores in the training dataset and test dataset. **(E, F)** Univariate Cox regression model and multivariate regression model with age, gender, stage, grade, T, N, M, and risk score in the training dataset and test dataset. Left, univariate Cox regression; right, multivariate Cox regression.

Survival curves, the distribution of patients’ status/survival time, univariate Cox regression, and multivariate Cox regression were conducted in the training dataset and test dataset to testify if the risk model was capable of predicting GC patients’ prognosis. Consistently, in both the two datasets, the OS of high-risk subgroups was shorter than that of low-risk subgroups (**Figures 4A, B**). The distribution of GC patients’ vital status and survival time according to the risk score is displayed in **Figures 4C, D**; the result demonstrated that samples in the low-risk subgroup had longer survival time than that in the high-risk subgroup, and there were more dead samples in the high-risk subgroup than in the low-risk subgroup. Meanwhile, whenever in the univariate Cox regression model or multivariate Cox regression model, the risk score could be recognized as an independent risk factor of GC patients in the training dataset and test dataset (**Figures 4E, F**). Furthermore, the ROC curves showed that the risk model had a promising capability to predict GC patients; the areas under the curve (AUCs) of 3-year OS in training and test datasets were 0.633 and 0.668 (**Figures S4A, B**); the AUCs of 5-year OS were 0.562 and 0.607, respectively (**Figures S4C, D**).

**Figure 5:**
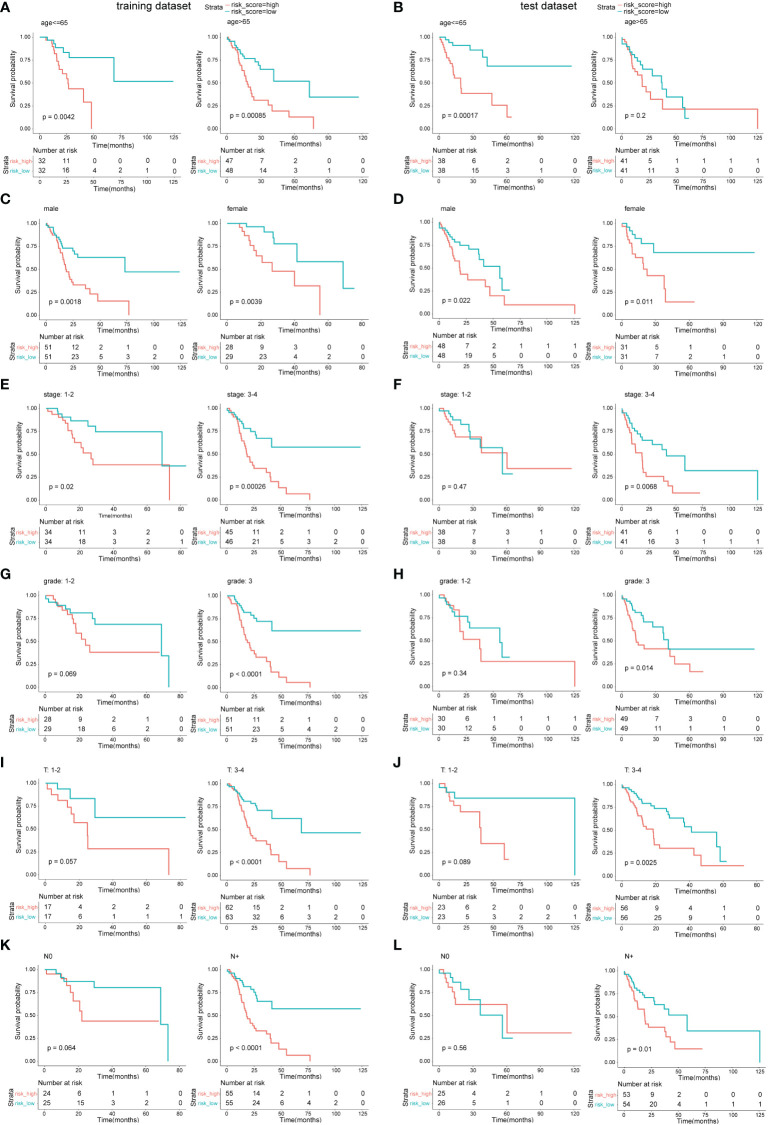
Survival analysis in different clinical feature cohorts. **(A, C, E, G, I, K)** OS comparison between the low-risk subgroup and the high-risk subgroup in different cohorts of the training dataset. **(B, D, F, H, J, L)** OS comparison between the low-risk subgroup and the high-risk subgroup in different cohorts of the test dataset.

To further validate the model, samples in the two datasets were grouped by clinical prognosis features including age, gender, disease stage, grade, and T and N stage. The OS curves between the low-risk subgroups and high-risk subgroups in the above cohorts are compared in **Figure 5**. The results indicated that in most of these cohorts, low-risk subgroups had longer OS than high-risk subgroups; however, in some cohorts, there was no statistical significance. These cohorts included stage 1–2 in the test dataset and grade 1–2, T 1–2, and N0 in both datasets.

### 3.4 Construction of nomogram model

**Figure 6:**
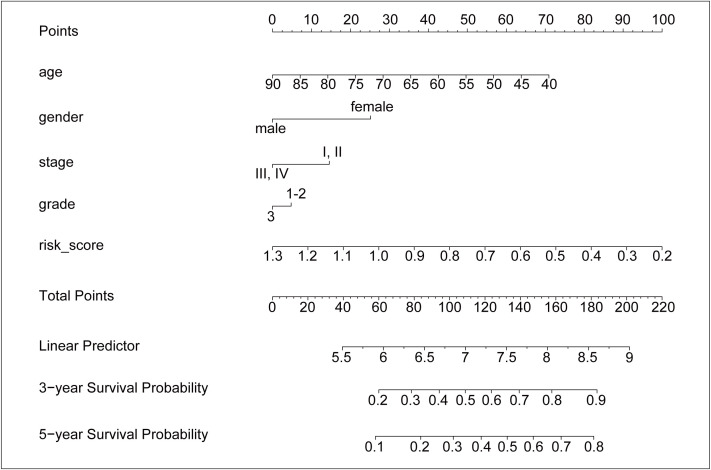
The nomogram for predicting the 3-year and 5-year survival probability of GC. The nomogram model was constructed in training dataset, with the age, gender, stage, grade, and risk score of six ALKBH5-related risk genes.

To obtain a quantitative tool for predicting the OS of GC patients, a nomogram model was built using age, gender, stage, grade, and risk score in the training dataset and was verified in the test dataset (**Figure 6**). In the training cohort, the calibration curves showed a strong and acceptable consistency of observed and predicted ratios in 3-year and 5-year OS, respectively (**Figures S5A, B**). The DCA curves of the nomogram indicated that if the threshold probability of 3-year OS was 0.16–0.39 and that of 5-year OS was 0.1–0.44, the nomogram could offer a higher net benefit than predicting for all patients or no patients (**Figures S5C, D**). These results of validation suggested that our nomogram had a strong ability and accuracy in predicting the OS of GC patients.

### 3.5 Relationship of the risk model and immune cell infiltration

**Figure 7:**
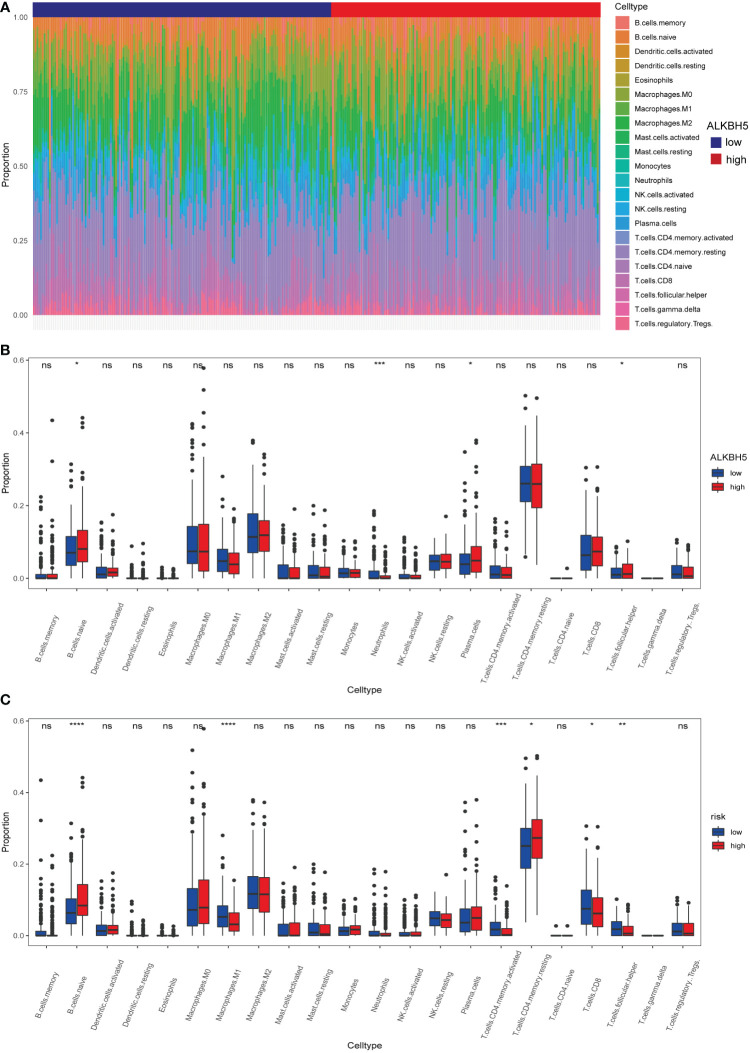
Different immune cells infiltration of *ALKBH5*-low/high subgroups and risk-low/high subgroups. **(A)** Relative proportions of 22 immune cells’ infiltration in each GC sample according to the expression of *ALKBH5*. **(B)** Comparison of 22 immune cell proportions infiltrated in GC samples between *ALKBH5*-low and *ALKBH5*-high subgroups in the whole dataset. **(C)** Comparison of 22 immune cell proportions infiltrated in GC samples between low-risk and high-risk subgroups in the whole dataset. ns, non-significance, *P<0.05, **P < 0.01, ***P < 0.001, ****P < 0.0001.

The DEGs might participate in various pathways to execute their functions. GO enrichment analysis, a method mainly used to perform enrichment analysis on gene sets (38), was carried out here to investigate the potential biological processes of these DEGs; the result indicated that some of the DEGs were enriched in biological processes such as the epidermis and the regulation of peptidase. Furthermore, part of them was involved in immunity activities such as the defense response (**Figures S6A, B**). Thereafter, the training dataset and test dataset were combined. The risk model was checked again in the whole dataset using the Kaplan–Meier method; the high-risk subgroup still led to poor OS (**Figure S4E**). Subsequently, the CIBERSORT algorithm (an analytical tool from the Alizadeh Lab developed by Newman et al. to provide an estimation of the abundances of member cell types in a mixed cell population, using gene expression data) was used to calculate the 22 immune cells infiltration proportion of each sample in the whole dataset (**Figure 7A**), results demonstrated that the ALKBH5-high subgroup was infiltrated with more naïve B cells, neutrophils, plasma cells, and follicular helper T cells (**Figure 7B**). Furthermore, the high-risk subgroup had more infiltration of naive B cells and resting CD4+ T cells, but the low-risk subgroup was infiltrated with more activated memory CD4+ T cells, CD8+ T cells, M1 macrophages, and follicular helper T cells (**Figure 7C**). The results indicated that the expression of *ALKBH5* shaped the immune conditions of tumor samples and, compared to the high-risk subgroup, the low-risk subgroup had a better immune microenvironment.

### 3.6 Validation of the lower ALKBH5 protein level in GC tissues and the change of related genes’ expression when ALKBH5 was knocked down

**Figure 8:**
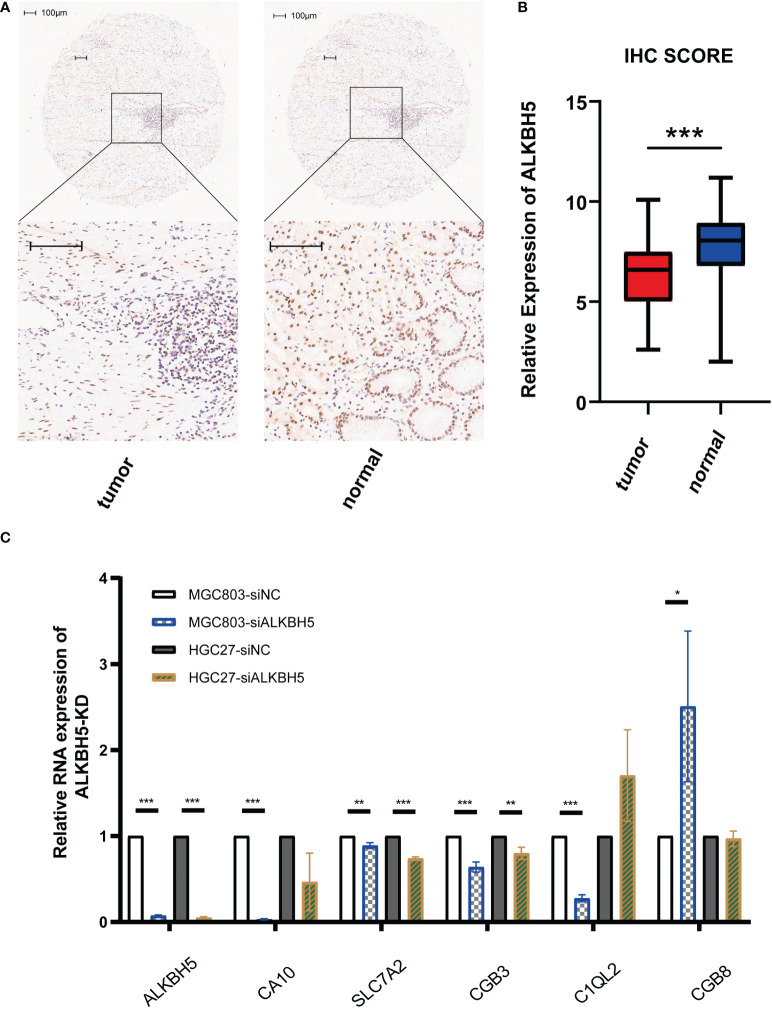
Validation of lowly expressed ALKBH5 in GC tissues and related genes’ mRNA-level response to *ALKBH5* knockdown. **(A)** Representative images of IHC for stained ALKBH5 in GC tumor tissues and adjacent normal tissues. **(B)** Box plot showing the IHC scores of the tumor group and normal group. **(C)** Relative RNA expression levels of related genes when *ALKBH5* was knocked down. Scale bar, 100 μm. *P < 0.05, **P < 0.01, and ***P < 0.001.

As protein is the main form for genes to lay functions, we detected the protein expression conditions of ALKBH5 using the IHC method in a GC tissue microarray. **Figures 8A, B** shows the representative images of IHC results and stained scores in GC tumor tissues and adjacent normal tissues; in line with the transcriptome profile in TCGA data, the protein expression of ALKBH5 was downregulated in GC tumor tissues. Moreover, to check if the genes used for constructing the risk were regulated by ALKBH5, we compared their relative mRNA expression levels between the si-NC and si-ALKHB5 groups. Results indicated that *SLC7A2* and *CGB3* were downregulated when *ALKBH5* was knocked down in both MGC-803 and HGC-27 cell lines, and other coding genes had different degrees of variation in one of the two cell lines along with *ALKHB5* knockdown (**Figure 8C**).

## 4 Discussion

In recent years, with the booming development of diagnosis technology and increased awareness of prevention, GC incidence has decreased rapidly, but mortality has been staying at a high level (1). For patients in advanced stages, surgery often fails to cure; furthermore, the functions of radiotherapy and chemotherapy in GC are limiting; thus, safe and effective therapeutic targets are much needed for each patient in advanced stages or had no sensitive drugs. After the birth of sequencing technology, especially next-generation sequencing (NGS) (40), the biological processes and modifications to regulate DNA, RNA, and proteins were increasingly discovered. As the most prevalent modification of RNA, m6A RNA methylation played important roles in various tumors, and the regulators of m6A have the potency to be a therapeutic target of GC.

In GC, a lot of research has revealed the important roles of m6A regulators, but previous studies mainly focused on the m6A methyltransferase complex at first, especially METTL3 (21–23). Although the relationship between “erasers” and patients’ prognosis or pathological features has been discovered more and more (26, 27), there are fewer reports about what roles ALKBH5 play in GC. Moreover, some researchers tried to identify the biomarkers for predicting the outcome of GC patients using bioinformatics methods. In the m6A field, the limit was that they integrated all of the m6A regulator genes or “erasers” for analysis (41, 42), which made it hard to tell which regulator is responsible and confused readers.

In present study, a new approach to predict GC patients’ prognosis based on ALKBH5 was established and testified. We discovered that *ALKBH5* was lowly expressed in GC tumor samples and it significantly decreased the OS of GC. In the training dataset, a risk model based on six ALKBH5-related genes was constructed. Multiple authentications in training/test datasets indicated that the high-risk subgroup led to poorer OS and the risk score seemed to be an independent risk factor of a GC prognosis in Cox regression models. Then, a nomogram model based on the risk score and other clinical features was built to predict the 3-year and 5-year OS of GC. GO and CIBERSORT analyses suggested that ALKBH5-related genes might be involved in the immune response and shaped the immune cell infiltration of GC samples.

After realizing the different expression levels of *ALKBH5* between tumor samples and normal samples, we first paid attention to it. The risk model and nomogram model were all derived from the DEGs between *ALKBH5*-low and *ALKBH5*-high subgroups; thus, in GC, not only was *ALKBH5* itself an independent protective factor but also its related genes had a probability to predict patients’ outcomes. This is the first model that came from a single gene, if the mechanism can be further illustrated, potential targets or therapeutic medicine might come true faster than those biomarkers derived from complex analysis. Furthermore, the six genes used for constructing the risk model were screened from DEGs by LASSO Cox regression, which could provide high prediction accuracy and prevent overfitting. In addition, the adenosine methylation sites in the six genes were predicted in the SRAMP database. Results suggested that there were a lot of sites with very high confidence or high confidence; these sites were the potential targets of ALKBH5.

In our study, there was an absorbing result. When validating the risk model in different clinical cohorts, the OS between low-risk and high-risk subgroups might have no significant difference in early stages, including stages 1–2 in the test dataset and grades 1–2, T 1–2, and N0 in both datasets. These results indicated that the risk model was more effective in advanced stages; thus, the six genes screened out might have participated in the critical biological process of GC development and had an important value in the therapy of advanced stage for GC patients. Moreover, this might be the possible reason for higher accuracy in predicting 3-year OS than 5-year OS because patients in advanced stages had shorter survival time.

Furthermore, the GO enrichment analysis in our study revealed that the DEGs related to *ALKBH5* were likely involved in the immune response. Immunotherapy and immune biomarkers exhibit an outstanding value in the diagnosis and therapy of tumors, which brought new hope to cancer patients including GC (43, 44). Immune activities are mainly executed by immune cells; the results in our study suggested that *ALKBH5* changed the immune microenvironment of GC by altering immune cells. Several reports had demonstrated that CD8+ T cells, M1 macrophages, and NK cells played an antitumor role in GC (45); resting memory CD4+ T cells were closely associated with the pathogenesis of GC (46). Moreover, the regulation of follicular helper T cells was critical to prevent autoimmunity in cancer (47). In present research, compared with the high-risk subgroup, samples in the low-risk subgroup were collected with more activated memory CD4+ T cells, CD8+ T cells, M1 macrophages, and follicular helper T cells, indicating a better microenvironment in the low-risk subgroup. Oppositely, there were more resting memory CD4+ T cells in the high-risk subgroup which led to a poorer prognosis. In addition, the proportions of neutrophils and plasma cells were higher in the *ALKBH5*-high subgroup. They emerged as significant but opposite predictors of survival for breast and lung adenocarcinomas (48); thus, the relationship between GC and them needed further investigation. Furthermore, CD4+ T cells and macrophages infiltrated in GC samples could decrease the tumor purity and low tumor purity in GC was associated with an unfavorable prognosis and the immune-evasion phenotype (49). Conclusively, while our results suggested that *ALKBH5* and its related genes might play an important role in the GC-immune microenvironment and could provide potential targets for immunotherapy in GC, careful consideration should be given before making immunotherapy decisions.

The IHC results were consistent with the transcriptome data in the TCGA database, indicating that the protein expression level of ALKBH5 in GC tumor tissues was lower than that in adjacent normal tissues. Moreover, compared with sequencing data, the protein staining in patients’ tissues was closer to the real world, which further confirmed lowly expressed ALKBH5 and the value of ALKHB5 as a prognostic marker in GC. In addition, although there were a lot of predicted m6A regulator sites on the six regulated genes used to build the risk model, how their expression changed the response to the altered expression of *ALKBH5* was not clear. qPCR results showed that when *ALKBH5* was knocked down, the mRNA levels of *SLC7A2* and *CGB3* were downregulated both in MGC-803 and HGC-27 cell lines, indicating that they are regulated by ALKBH5. While there was no distinct relation between these two genes and of GC, *SLC7A2* has been proven to be lowly expressed in hepatocellular carcinoma (HCC) and suppress the progress of HCC (50). Furthermore, *CGB3* also acts as a tumor suppressor in cervical cancer (51). *ALKHB5*, *SLC7A2*, and *CGB3* showed a similar tendency to inhibit tumor development, suggesting that *SLC7A2* and *CGB3* may also play a suppressor role in GC; the internal mechanism of how *ALKBH5* regulates these genes needs to be further explored.

Although the present study gave prospective new signatures in GC, there remain some limitations. Firstly, the risk model is mainly from bioinformatic analysis, while the tumor and cells are not in computer and *silico*, it is needed to validate the function of these genes and the immune cell infiltration *in vitro* and *in vivo.* Secondly, as a retrospective study, there must be selection bias in this study; more sequencing data, especially those hospitals, should be adopted for further analysis. Thirdly, based on the transcriptome, IHC, and qPCR analysis, the functions and mechanism of these signature genes should be investigated thoroughly in the future.

In summary, our results suggested that *ALKBH5* was lowly expressed in GC and played a role as a repressor. We screened the DEGs in *ALKBH5*-low subgroup/*ALKBH5*-high subgroup and got 6 genes (*CA10*, *SLC7A2*, *LINC02303*, *CGB3*, *C1QL2*, *CGB8*) to construct a risk model by LASSO regression. The risk model and nomogram model were validated and showed promising ability for predicting the prognosis. Furthermore, ALKBH5 and its related genes could alter the proportion of immune cell infiltration and provide potential targets for immunotherapy of GC. In addition, the low protein expression of ALKBH5 in GC tissues and its simple regulation of the risk model related genes were checked. Findings in this study suggest that ALKBH5 may be a suppressor of GC, ALKBH5 and its related genes have the probability to be markers to indicate the progression and immunotherapy end of GC.

## Data availability statement

The original contributions presented in the study are included in the article/**Supplementary Material**. Further inquiries can be directed to the corresponding author.

## Ethics statement

The studies involving human participants were reviewed and approved by Ethics Committee of Shanghai Outdo Biotech Co., LTD Shanghai Outdo Biotech Company. The patients/participants provided their written informed consent to participate in this study.

## Author contributions

TJ, XG and JL conceived and designed the study. XG and DL refined the research design idea. TJ and XG downloaded the data. TJ and YC the data. XG and DL accomplished the RT-qPCR assay. DL, SH and XA further supplemented and validated the data and assisted with the interpretation of the results. TJ, XG and DL drafted the manuscript. SH prepared **Figure 1**. YC and XA revised the logic of the manuscript and polished the language. WJ and SY accomplished the IHC assay, corrected spelling mistakes and sorted out the references. JL revised and proofread the manuscript. All authors contributed to the article and approved the submitted version.
