# Supplementary material for: Identification and validation of signature for prognosis and immune microenvironment in gastric cancer based on m6A demethylase ALKBH5
Source: Front Oncol. 2023 Jan 6;12:1079402. doi: 10.3389/fonc.2022.1079402 (PMC9853004; doi:10.3389/fonc.2022.1079402)

Figure S1. The expression between ALKBH5-low and ALKBH5-high subgroups in GSE29998.

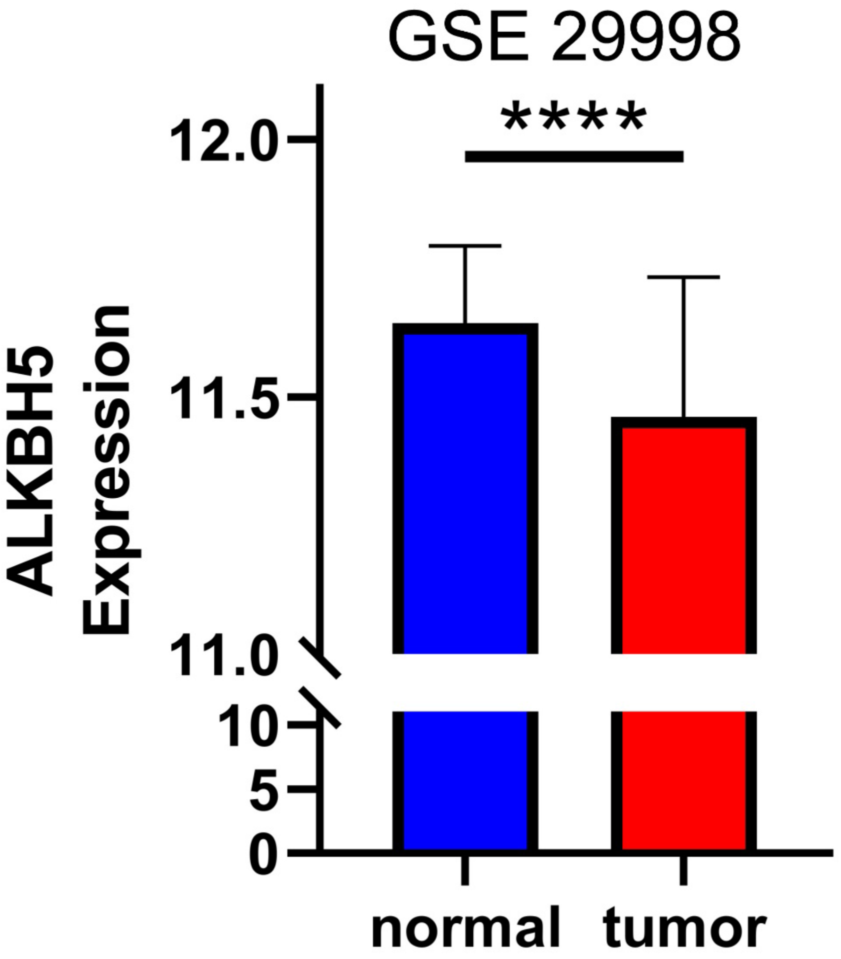

Figure S2. Univariate Cox regression and multivariate Cox regression of m6A regulated genes and clinical factors.

(A) Univariate Cox regression. (B) Multivariate Cox regression of significant ones in (A).

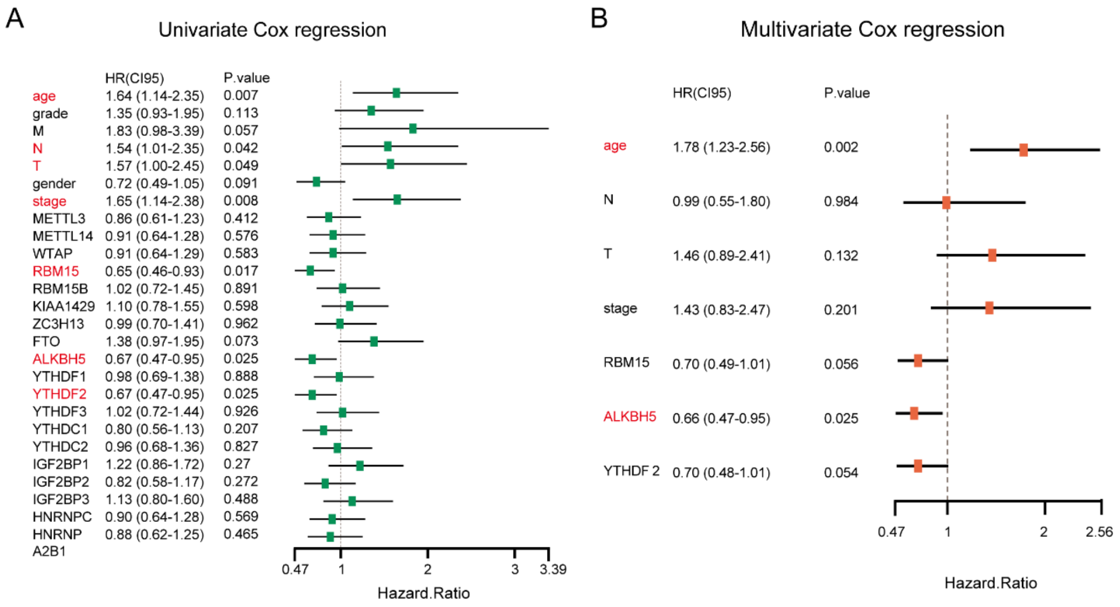

Figure S3. Prediction of adenosine methylation sites in the SRAMP database. Black arrows, sites with very high confidence.

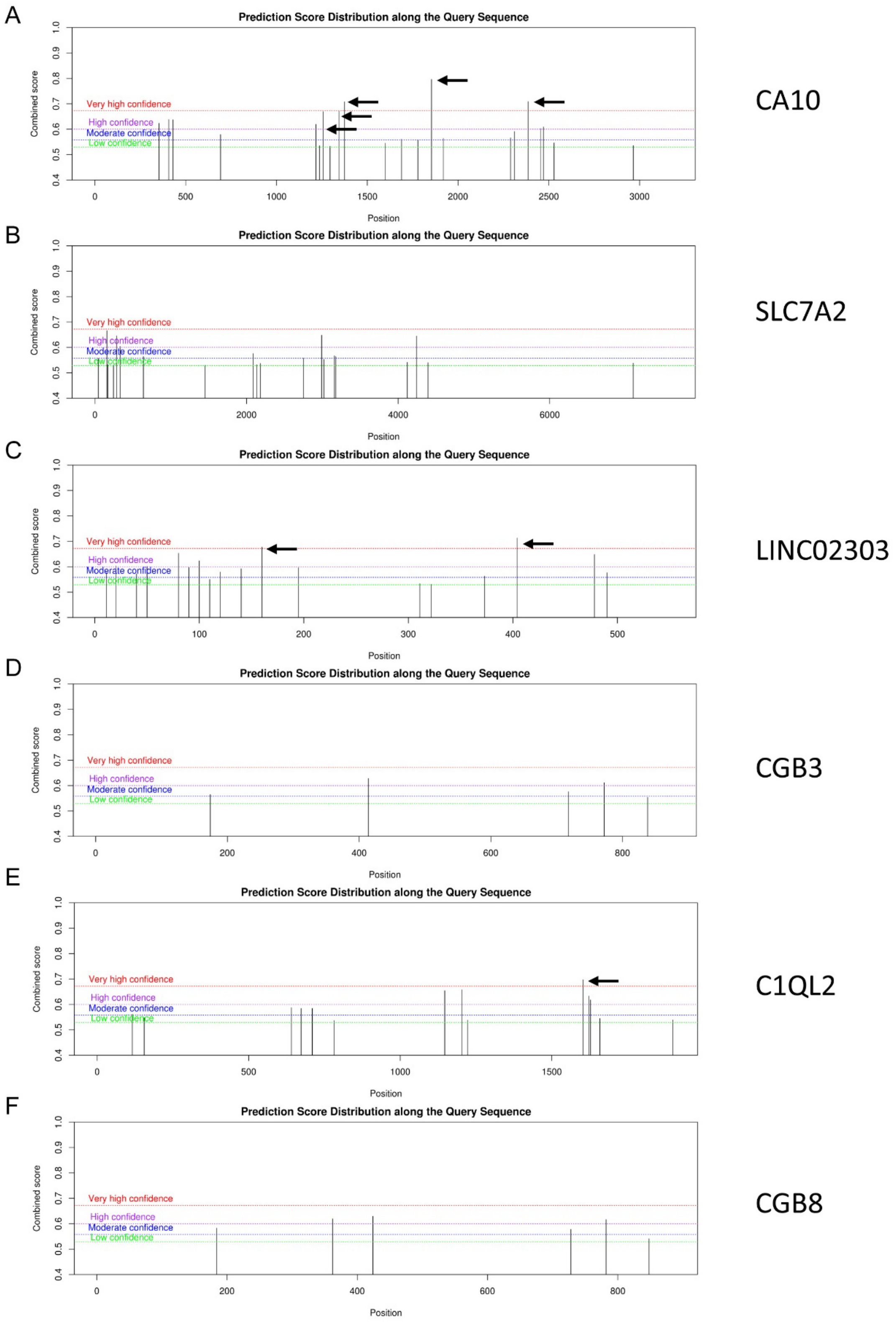

Figure S4. ROC of risk score in training/test datasets and OS in whole dataset.
(A) ROC of risk score for 3 years survival in training dataset. (B) ROC of risk score for 3 years survival in test dataset. (C) ROC of risk score for 5 years survival in training dataset. (D) ROC of risk score for 5 years survival in training dataset. (E) OS of high-risk subgroup and low-risk subgroup in whole dataset.

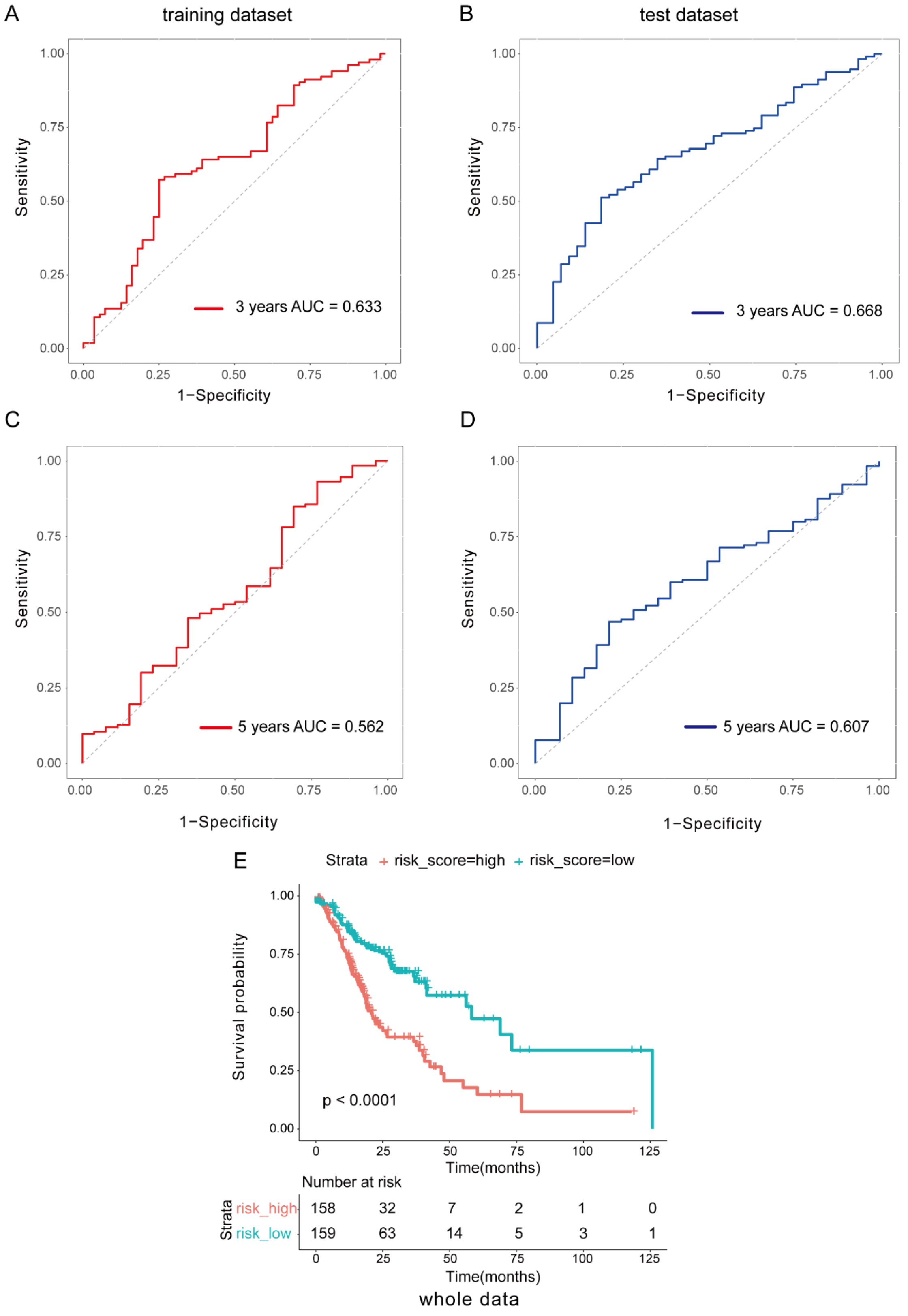

Figure S5. The accuracy and clinical utility of nomogram in prediction of 3-year and 5-year OS probability in gastric cancer.

(A) The calibration curve of the model based on the consistency between predicted and observed 3-year OS in training dataset. (B) The calibration curve of the model based on the consistency between predicted and observed 5-year OS in training dataset. Close-ended vertical lines, 95% CIs. (C, D) Decision curve analysis (DCA) used to assess the clinical utility of the nomogram. The x-axis represents the percentage of threshold probability and the y-axis represents the net benefit.

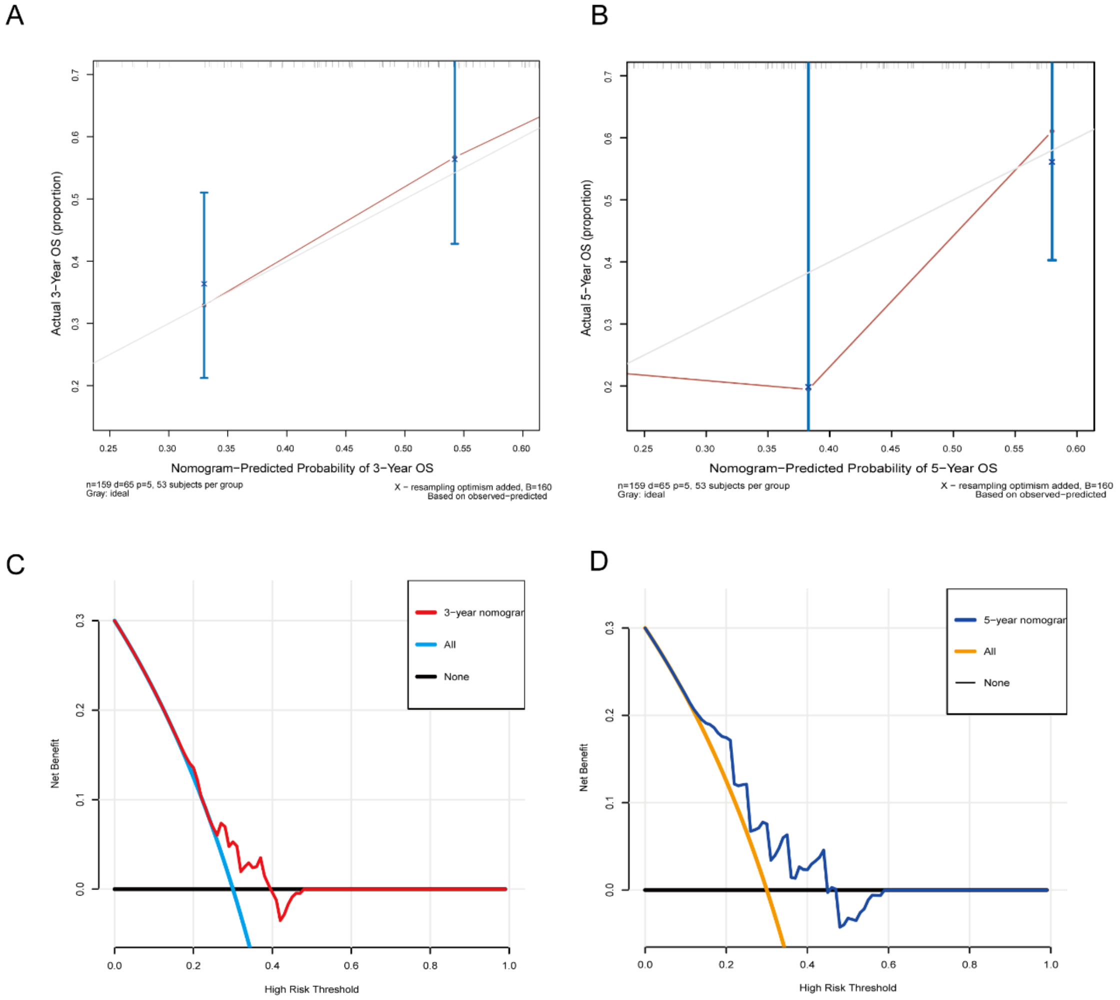

Figure S6 GO enrichment analysis of DEGs.

(A) GO analysis of DEGs in “molecular function”, “cellular component”, and “biological process” manners. (B) GO analysis of DEGs in “biological process” manners, showed with p-value and counts. Red frames, biological processes about immune response.

A

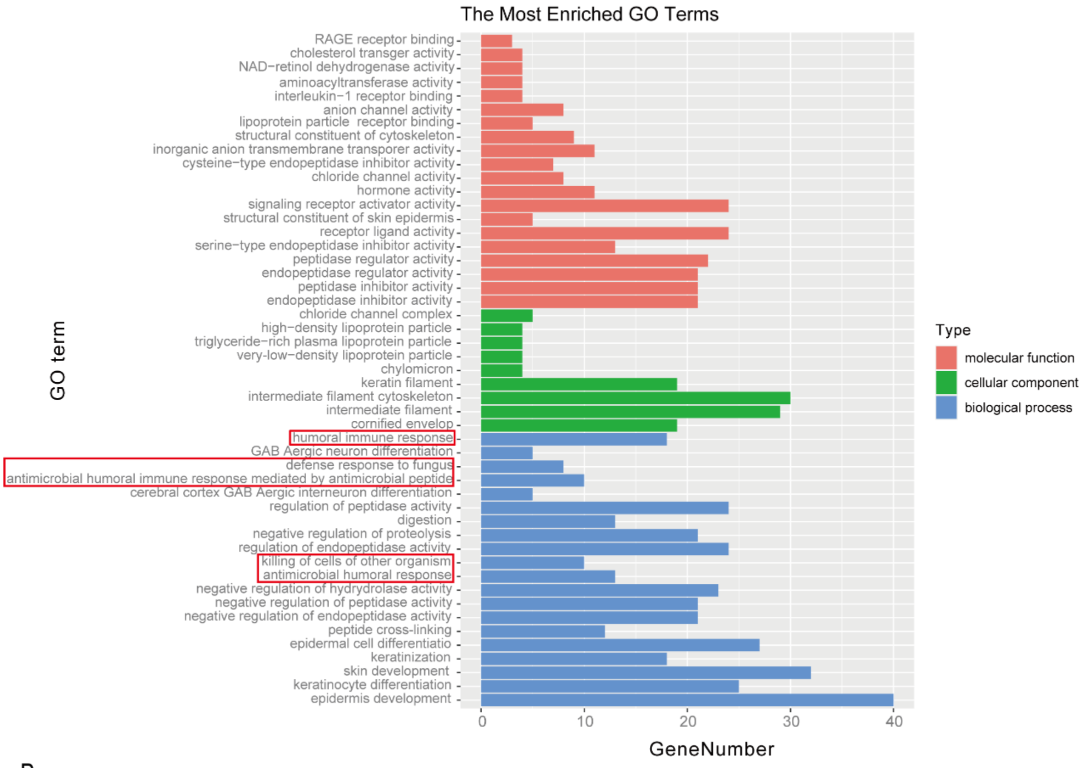

B

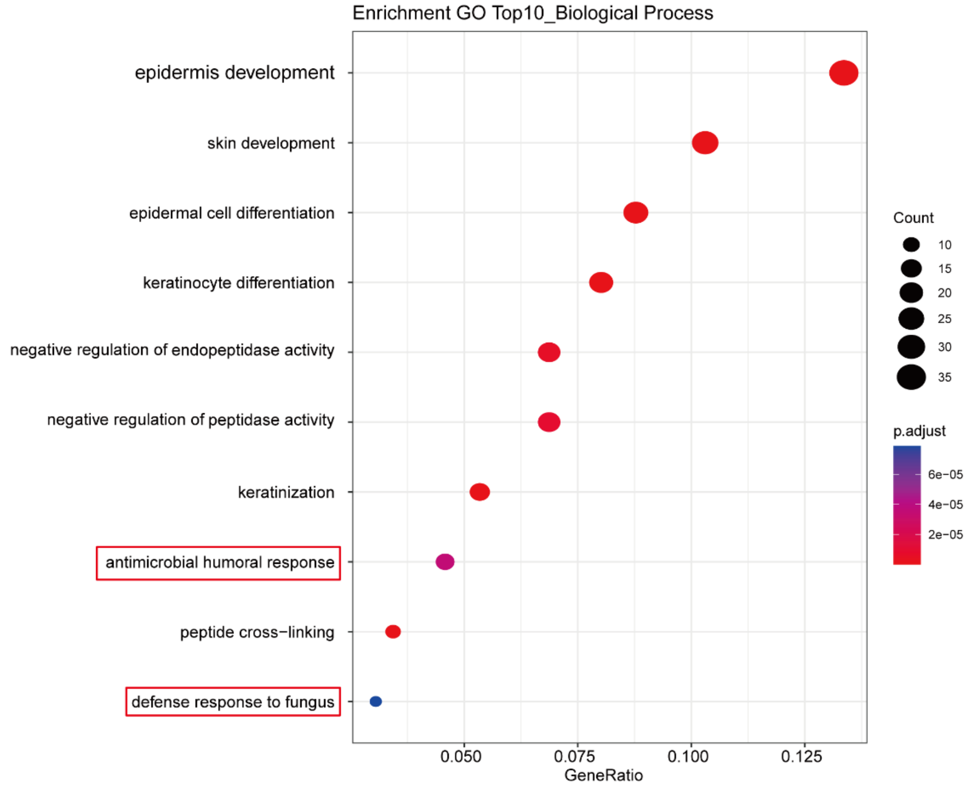

Supplement: Supplementary file 1 [file Image_1.pdf]
